# Effect of Citrullus lanatus juice in human sperm

**DOI:** 10.5935/1518-0557.20240002

**Published:** 2024

**Authors:** Walter D Cardona-Maya

**Affiliations:** 1 Reproduction Group. Department of Microbiology and Parasitology. Faculty of Medicine, Universidad de Antioquia. UdeA, Medellin, Colombia

Dear Editor

I read the recently published review article by Damilare E. Rotimi and Rotdelmwa M. Asaleye entitled “Impact of Watermelon (*Citrallus lanatus*) on Male Fertility” (Rotimi & Asaleye, 2023).

This exciting review, after reviewing the available data, demonstrates that watermelons (*Citrullus lanatus*) improve male fertility because the evidence in animal models shows that it improves seminal quality, reverses erectile dysfunction, and improves testicular redox status and gonadotropin secretion; however, no evidence of this improvement in humans was included.

In 2020, our group published a paper that sought to evaluate the effect of watermelon juice on conventional and functional seminal parameters *in vitro* and *in vivo* (Saldarriaga Monsalve & Cardona Maya, 2020). In this work, during *in vivo* assays, conventional and functional sperm parameters were determined on days 0, 7 and 15 after starting daily consumption of 16 ounces of watermelon juice in 20 individuals. We found that regular watermelon consumption juice decreases sperm membrane lipoperoxidation, intracellular production of reactive oxygen species, DNA fragmentation index on day 15 and antioxidant capacity on days 7 and 15. In addition, during the in vitro process, five pure sperm samples were incubated with hydrogen peroxide (H_2_O_2_, 5mM) and 0.45% watermelon extract, and it was observed that the extract protected the harmful effect of H_2_O_2_ on sperm motility.

Consistent with these results, a previous study showed that pre-incubation of human spermatozoa with lycopene protects them against DNA damage in samples incubated with H_2_O_2_ (Zini *et al*., 2010).

In watermelon the percentage of lycopene in watermelons, a powerful antioxidant, comprises 70-90% of the total carotenoids, averaging an average concentration of 4868µg/100g (Edwards *et al*., 2003). Therefore, regular consumption of watermelon could improve the functional seminal parameters related to reproductive success; as seen in the results obtained in vitro, watermelon generates a protective effect on human sperm *in vitro*, protecting sperm motility from the negative effect of H_2_O_2_.

This evidence supports an attempt to elucidate some possible mechanisms of the positive effect of watermelon consumption and emphasizes, as highlighted in the literature review, the importance of clinical studies with large sample sizes, varied duration of administration, comparison with safe drugs and determination of the exact molecular mechanism.
